# Hippocampus RNA Sequencing of Pentylenetetrazole-Kindled Rats and Upon Treatment of Novel Chemical Q808

**DOI:** 10.3389/fphar.2022.820508

**Published:** 2022-03-08

**Authors:** Xiang Li, Qing Wang, Dian-wen Zhang, Di Wu, Si-wei Zhang, Zheng-ren Wei, Xia Chen, Wei Li

**Affiliations:** ^1^ Department of Pharmacology, College of Basic Medical Sciences, Jilin University, Changchun, China; ^2^ Jilin Provincial Academy of Traditional Chinese Medicine, Changchun, China

**Keywords:** Q808, novel anti-convulsant chemical, PTZ-kindled seizure, RNA sequencing, hippocampus

## Abstract

The expression of genes altered in epilepsy remains incomplete, particularly in the hippocampus, which exhibits exquisite vulnerability to epilepsy. Q808 is an innovation chemical compound that has potent anti-convulsant effect. Exploring its mechanism can not only explore the pathogenesis of epilepsy but also provide a theoretical basis for its clinical application. The present study aimed to use RNA sequencing (RNA-seq) to reveal the gene transcriptomic profile of chronic pentylenetetrazole (PTZ)-kindled seizure rats and the difference of the PTZ model rat before and after treatment with Q808. Quantitative real-time PCR (qRT-PCR) was performed to validate the RNA-seq results. The protein level was estimated with Western blot. Hippocampal transcriptomic analysis showed that 289 differentially expressed genes (DEGs) were confirmed in the PTZ-kindled seizure group compared with the vehicle control. Gene cluster analysis identified most of the DEGs linked to neuronal apoptosis, neurogenesis, neuronal projections, and neurotransmitter regulation. After analysis across the three groups, 23 hub genes and 21 pathways were identified, and qRT-PCR analysis confirmed that most of the mRNA levels of hub genes were consistent with the RNA-seq results. Q808 treatment increased the level of ACE, a GABA-related protein. Our analysis showed the comprehensive compendium of genes and pathways differentially expressed for PTZ-kindled seizure rats and upon Q808 treatment in PTZ-kindled seizure, which may provide a theoretical basis to explore the mechanism and unique efficacy of Q808 and the pathophysiology of epilepsy in the future.

## Introduction

Epilepsy is a relatively common neurological disorder that affects people of all ages. Approximately 2–3% of the population worldwide have epilepsy (Gavvala and Schuele, 2016). The typical symptoms of epilepsy include recurrent unprovoked seizures and psychiatric symptoms, such as fear and anxiety, which considerably diminish the quality of life of a person (Thijs et al., 2019). The pathophysiology of epilepsy has various mechanisms, including neurotransmitter imbalance, channelopathies, neural migration, neuronal loss, brain injuries, degenerative disorders, morphological abnormalities, cortical and/or hippocampal malformations, and genetic background (Brodie et al., 2016; Pfisterer et al., 2020). Several anti-epileptic drugs are available in the market, but about one-third of the epileptic patients fail to achieve seizure control, and they develop refractory epilepsy (Löscher et al., 2013). In addition, traditional anti-epileptic drugs show various and serious adverse reactions (Stefan and Feuerstein, 2007). Therefore, identifying a new anti-epileptic agent is necessary.

**FIGURE 1:**
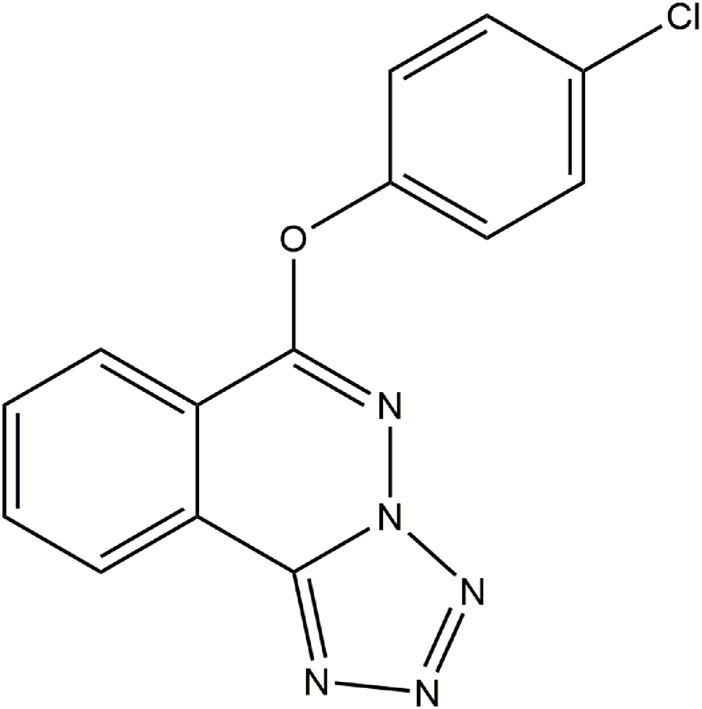
Chemical structure of Q808.

Q808, 6-(4-chlorophenoxy)-tetrazolo[5,1-a]phthalazine (Figure 1), is an innovative chemical component with an international patent, which showed a broad-spectrum anti-epileptic activity in various experimental seizure models including a maximal electroshock seizure model and chemical-induced seizure model, such as induced by PTZ, 3-mercaptopropionic acid (3-MP), thiosemicarbazide (THIO), and isoniazid (ISO) (Sun et al., 2010). In our previous study, we found that Q808 can specifically increase the level of GABA, an inhibitory neurotransmitter, and enhance the frequency of spontaneous inhibitory postsynaptic currents in the hippocampus (Li et al., 2017). However, knowledge about the molecular mechanisms of Q808 is still limited.

The hippocampus is a heterogeneous structure with distinct regional connectivity along its longitudinal axis, and it plays a significant role in epileptogenesis and seizure maintenance (Strange et al., 2014; Huberfeld et al., 2015). The chemical kindling of seizures triggered by PTZ has been used to reflect the pathogenesis of human epilepsy, which is considered an appropriate model for drug-resistant epilepsy (Löscher, 2017). In the present study, hippocampal damage, such as neuronal apoptosis, has been observed after PTZ-induced seizure (Taskıran et al., 2020). This damage in the hippocampus can not only promote the occurrence and development of epilepsy but also cause impairment of cognitive function. Sections from PTZ-treated seizure mice showed morphological changes such as vacuolation and several degenerative areas with darkly stained nuclei in the hippocampus (El-Missiry et al., 2020). Apart from the change of neuro, some neurotransmitters also significantly change in the hippocampus of the PTZ model. In our previous study, inhibitory neurotransmitters such as GABA decreased evidently in the hippocampi of the PTZ-kindled model (Li et al., 2017). These changes in hippocampus could be associated with the genetic change. Considerable evidence has shown that the mRNA expression levels of the subunit of the GABAA receptor and apoptotic genes, namely, *Bax* and *Caspase-3*, were significantly increased (Ahmadirad et al., 2014; Singh et al., 2019). Thus, characterizing the gene expression profiles of the hippocampus is crucial to elucidate the molecular mechanisms of epileptogenesis and the anti-epileptic effect of Q808.

RNA-seq is a novel and sophisticated approach to comprehensively understand the molecular mechanisms of epilepsy in recent years (Chen et al., 2020). In the previous study, transcriptomic analyses have been performed with several seizure models, such as human intractable partial epilepsy (Guelfi et al., 2019), human temporal lobe epilepsy (Conte et al., 2020), drug refractory epilepsy patients (Dixit et al., 2016), and mouse and rat kainic acid models (Dong et al., 2020; O'Leary et al., 2020). An intraperitoneal continuous low-dose injection of PTZ into an animal could induce a severe tonic–clonic seizure, and this kindling model is widely applied to investigate the pathophysiology of epilepsy and epilepsy-related genes (Shimada and Yamagata, 2018). However, no studies have reported alterations in the hippocampal transcriptome of the PTZ-kindled seizure model before and after treatment with Q808.

In the current study, the chronic PTZ-kindled seizure model and seizure model treated with Q808 for 4 weeks were used, and the anticonvulsant effect of Q808 on the PTZ-kindling model was confirmed. In addition, RNA-seq was used to investigate the alternations in mRNA expression profile in the rat hippocampus. Furthermore, differential gene expression and level of a GABA-related protein were validated. The results presented in the study may benefit identifying novel mechanisms for epilepsy.

## Materials and Methods

### Chemicals

The Q808 compound was a kind gift from the Academy of Chinese Medical Sciences of Jilin Province, and the synthesis conformation of Q808 has been verified (Zhang et al., 2009). The other drugs and reagents, such as PTZ, Tween-80, and CMC-Na, were purchased from Sigma-Aldrich Chemical Company (St. Louis, United States). ACE, β-tubulin, and Goat Anti-Rabbit IgG (H + L) HRP antibody were purchased from Affinity Biosciences (Affinity, United States).

### Experimental Animals

Experimental procedures were carried out in accordance with the National Institutes of Health guidelines and approval by the Animal Care and Use Committee of the College of Basic Medical Sciences in Jilin University (No. 2020-24). Thirty specific pathogen–free Wistar rats (180–220 g) were purchased from Changchun Yisi Experimental Animal Technology Co., Ltd. (Changchun, China; certificate number SYXK 2018-0001). The rats were housed three per cage under controlled temperature (22–25°C) and humidity (50–60%) with a 12°h light/dark cycle. Rats had free access to water and food. One week later, the animals were randomly divided into the vehicle control group (Con.), PTZ + vehicle group (PTZ), and PTZ + Q808 group (Q808). Rats in the PTZ group and Q808 group received intraperitoneal (i.p.) injection of sub-convulsive dose (35 mg/kg) of PTZ once every 48 h for the first 28 days (14 injections) and 60 mg/kg of PTZ on the last day of treatment to achieve a fully kindled state (Mahmoudi et al., 2020). The Con. group received equal amounts of normal saline. For the next 28 days, the Q808 group received Q808. The Con. group and PTZ group received the solvents of Q808 at the same time and same route as the Q808 group.

### Behavioral Assessment of Seizure

The PTZ-kindled seizures were scored according to a modified Racine scale (Racine, 1972): category 0, no evidence of convulsive activity; category 1, whisker trembling and mouth and facial twitch; category 2: head nodding; category 3: forelimb clonus; category 4: forelimb rearing or jumping; and category 5: falling and tonic–clonic seizures. Rats with at least three consecutive seizures that scaled greater than or equal to Racine category 3 within the first 28 days were considered successfully kindled (Löscher, 2017) (success rate, 70%). The seizure latency to category 2 was also recorded.

### Drug Treatment

Q808 drug solutions were freshly prepared with Tween-80 and 0.5% CMC-Na, and 30 mg/kg was administered to the rats via oral gavage daily for 28 days. The dose, route, and timing of administration of Q808 were based on pharmacokinetic considerations and preliminary experiments (Zhang et al., 2009; Sun et al., 2010; Li et al., 2017). The body weights of the experimental animals were measured every other day. On the next day of the last injection of Q808, animals in PTZ and Q808 groups were injected with a sub-convulsive dose of PTZ. Behavioral assessment of seizure was done as described above.

### Hippocampal Tissue Preparation

The experimental animals were euthanized by decapitation under light ether anesthesia after the last seizure evaluation. Brains were removed and washed in cold phosphate-buffered saline, meninges were removed, and the hippocampus was bilaterally dissected on an ice platform. Individual samples were collected and stored in liquid nitrogen.

### RNA Extraction and Library Preparation

Total RNA was extracted from the hippocampal tissue sample (about 100 mg) using the TRIzol kit (Invitrogen, United States) according to the manufacturer’s instructions. RNA purity was checked, and quantification was evaluated using the NanoDrop 2000 spectrophotometer (Thermo Fisher Scientific, United States). Samples with an absorbance (260/280) ratio between 1.8 and 2.2 were considered acceptable. An Agilent 2100 Bioanalyzer (Agilent Technologies, United States) was used to assess the RNA integrity. Then, the sequencing library was generated by using the MGIEasy Universal Library Convert Kit (MGI, China), VAHTS^®^ Universal V6 RNA-seq Library Prep Kit for Illumina (Vazyme, China), and VAHTS^®^ mRNA Capture Beads (Vazyme, China) according to the manufacturer’s protocols.

### RNA Sequencing and Data Analysis

The libraries were sequenced on a BGI T7 platform. Raw reads were generated first and processed using the Trimmomatic software to remove reads containing adapter and ploy-N or low-quality reads. After this step, clean reads were obtained. By using Cufflinks (Trapnell et al., 2010), fragments per kilobase of transcript per million fragments mapped (FPKM) of each gene was calculated. HTSeq-count (Anders et al., 2015) was used to obtain the read counts of each gene. Differentially expressed gene (DEG) analysis was performed using the DESeq (2012) R package. The threshold for significantly differential expression in each sample was set at *p* value <0.05, and log2 of the fold-changes was filtered at > 1.5 or <0.67. In addition, hierarchical cluster analysis of DEGs across the three groups was performed to explore transcripts’ expression pattern.

### Enrichment Analysis

Gene Ontology (GO) and Kyoto Encyclopedia of Genes and Genomes (KEGG) enrichment analyses are often used to perform the functional annotation of DEGs. In detail, GO analysis was usually used to evaluate the biological process, cellular component, and molecular function of DEGs (Ashburner et al., 2000). The annotations were obtained from Gene Ontology (www.geneontology.org/) and NCBI (www.ncbi.nlm.nih.gov/). For identifying the pathways which DEGs are involved in, KEGG pathway analysis was used according to the KEGG database (www.genome.jp/kegg/) (Draghici et al., 2007). The two above-mentioned enrichment analyses were performed using R based on the hypergeometric distribution. A value of false discovery rate (FDR) less than 0.05 was considered significant.

### qRT-PCR Validation

RNA utilized for qRT-PCR was extracted with TRIzol as described above. First-strand cDNA synthesis was performed using the Prime Script^TM^ RT Master Mix (Takara Biological Engineering Company, China). Real-time PCR amplifications were performed in the QuantStudio Real-Time PCR System with QuantStudio design and analysis software in a 20-μl reaction volume. Each reaction tube contained 2 μl cDNA, 10 μl SYBR Green Master Mix (Roche, Switzerland), 1.25 μmol.L^−1^ forward and reverse primers, and RNase-free water. The cycling parameters were as follows: an initial hold stage of 95 °C for 10 min followed by 60 cycles of 95°C for 15 s and 59°C for 30 s. The target mRNA levels were normalized to the β-actin internal control gene. The fold-change in mRNA expression was analyzed according to the 2^−△△CT^ method. Primers for selected genes were designed using Primer-BLAST (Primer3 Input version 0.4.0 and BLAST) and are listed in Table 1.

**TABLE 1:** Sequences of the primers.

Gene name	Sequence (5′→ 3′)		GenBank accession
	Forward	Reverse	
*Ace*	GAT​TGC​AGC​CGG​GCA​ACT​TT	CTC​CGT​GAT​GTT​GGT​GTC​GT	NM_012544
*Alas2*	GCC​AAT​CAG​TGC​CTC​GTT​TC	CGG​TAT​GTG​TGG​TCC​TGC​TT	NM_013197
*Ccdc88b*	GAG​AGC​AGC​GAG​AGT​ACC​TG	ACG​AGG​ACC​CTG​TCC​CAT​C	XM_215206.11
*Col18a1*	GAA​GCT​CCG​TGG​CTC​TCT​AC	GGG​GGT​TTT​GCG​TAC​TCT​CA	NM_053489
*Doc2a*	CTC​GGA​TGA​TAC​CAC​TGC​CC	ACC​TTG​TGG​GTG​ATG​TCG​TC	NM_022937
*Enpp2*	TGT​TCG​TCC​TGA​TGT​CCG​TG	TTG​AAG​GCG​GGG​TAC​ATT​GG	NM_057104
*Faap100*	GCT​GCA​TAC​AAC​CTT​GCA​CC	CAC​ACA​GGA​CGT​AGA​GTG​CC	XM_039087819.1
*Fbl*	GTT​GAG​TTC​TCC​CAC​CGC​TC	AAA​GTG​TCC​TCC​ATT​CCG​CA	NM_001025643
*Fhl2*	GAC​TGA​ACG​CTT​TGA​CTG​CC	CCA​GTG​GCG​ATC​CTT​GTA​GG	NM_031677
*Gypc*	CTC​AGT​GTG​TGT​GAG​GTG​GTT	CAT​CTT​GAG​CTG​CGT​TGA​TGG	NM_001013233
*Hbb*	TTG​TGT​TGA​CTC​ACA​AAC​TCA​GAA	GCC​ACC​AAC​ATC​ATC​AGG​GTT	NM_033234.1
*Itpripl1*	GCC​TCC​TGT​AGT​TTG​TGC​GA	TAA​AGA​GAC​AGC​GCG​ACA​GG	NM_001025043
*Lmo4*	TCG​GGC​TGA​GAC​TTG​ACA​CTT	CCC​TAT​ATT​GCA​ACA​CTG​CGA	NM_001009708
*Man2b2*	CGT​CCA​GTG​TCA​GCA​CAG​AT	TGT​GCG​CCT​GAC​TTT​GTA​GT	NM_001134971
*Mex3a*	GAG​CGA​CCA​TCA​AAC​GCA​TC	GGT​GAT​CTC​AAA​CAC​GGG​GT	XM_039103755.1
*Mvd*	AGG​GGT​GTC​TCC​TGT​GAT​GA	GCC​CTC​ATC​CTT​CAC​GCT​TA	NM_031062
*Ovol2*	TCT​CAC​GAC​ACC​CTA​ATG​AGC​C	TTT​TTG​ACC​TGG​CAA​CCG​GG	NM_001106519
*Shox2*	CGC​AAA​GAG​GAT​GCG​AAA​GG	AAC​CAA​ACC​TGT​ACT​CGG​GC	NM_013028
*Slc25a22*	CCC​ATC​GAC​CTG​GCT​AAG​AC	CCA​GCT​TGA​TGG​CCT​TCT​CA	NM_001014027
*Sox11*	CGT​GGC​GGT​CAG​GAT​AAA​GA	GGG​TGG​GGG​AAA​TCA​CAA​CT	NM_053349
*Wdr54*	TCA​CAG​CCA​CAC​CTC​TAA​ACA	CTG​CCC​TAC​CTC​CCT​ACA​CTA	NM_001109245
*β-Actin*	AGG​CCC​CTC​TGA​ACC​CTA​AG	CCA​GAG​GCA​TAC​AGG​GAC​AAC	NM_001199954

### Western Blot

Total protein was extracted from the hippocampal tissues with radio immunoprecipitation assay lysis buffer at 4°C for 30 min, and the total protein concentration was quantified by a BCA Protein Assay Kit (Solarbio, China). Equal amounts of protein were loaded on 8% sodium dodecyl sulfate–polyacrylamide gel and then transferred to polyvinylidene fluoride membranes with ice cooling. After blocking with 5% non-fat milk, the membranes were incubated with primary antibodies, rabbit Anti-ACE1 (1:1,000, Affinity), overnight at 4°C. After washing, membranes were soaked in the secondary antibodies, Anti-rabbit IgG HRP-linked (1:5,000, Affinity), for 1 h at room temperature. β-Tubulin was used as an internal control. Images were visualized by enhanced chemiluminescence using Tanon-4200SF (Biotanon) and analyzed using ImageJ software.

### Statistical Analysis

The GraphPad Prism 7 software was used for data analysis. Data were expressed as mean ± SD. Statistical analysis was carried out by the Mann–Whitney U test and unpaired t-test to compare the difference in seizure score (a discrete variable) and seizure latency (a continuous variable), respectively, between two groups. One-way ANOVA followed by Tukey’s test was used to compare differences in hub gene and protein expression among three groups, and the experiment was performed with at least three biological replicates. An adjusted *p*-value < 0.05 was considered to be statistically significant.

## Results

### Racine Score and Seizure Latency

**FIGURE 2:**
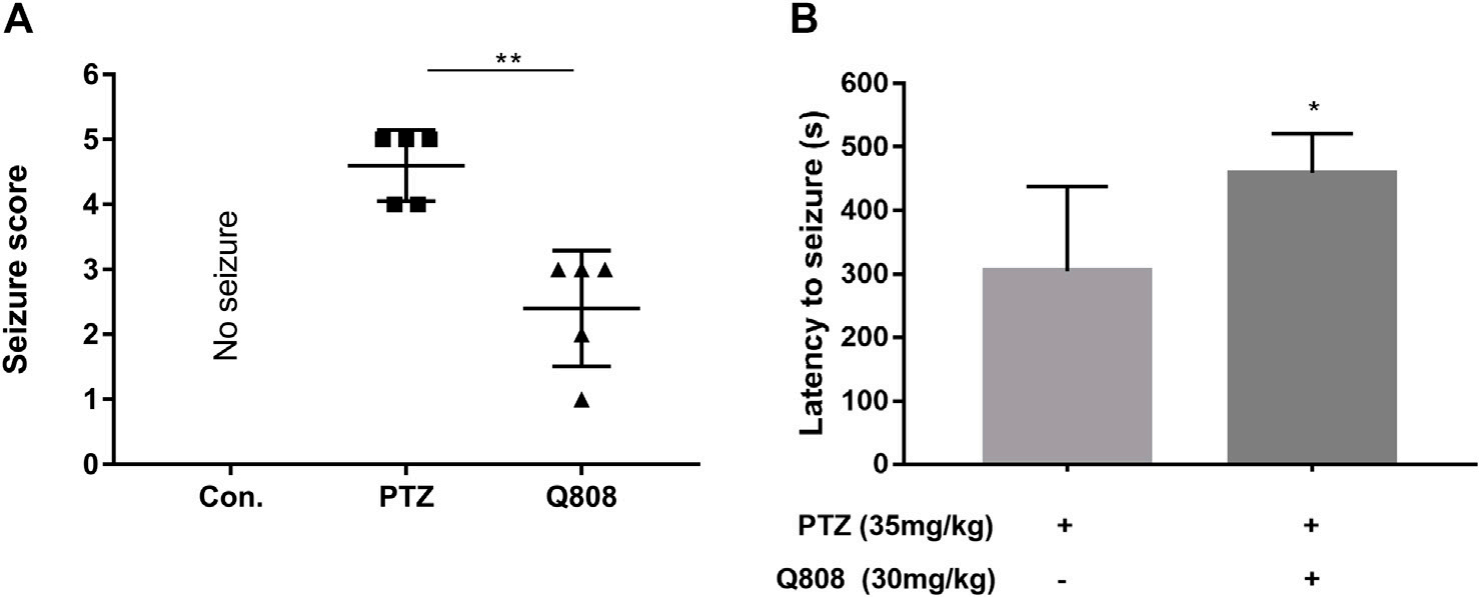
**(A)** Seizure score and **(B)** latency to seizures of PTZ-kindled rats before and after treatment with Q808. Data are shown as mean ± SD. Statistical analysis was carried out by the Mann–Whitney U test and unpaired t-test, respectively. *n* = 5, *p < 0.05, and **p < 0.01.

A sub-convulsant dose of PTZ was intraperitoneally injected once every 2 days for the first 28 days of the seizure model. Then, Q808 was given via oral gavage daily for the following 28 days. Behavioral changes were evaluated at the end of the drug treatment. PTZ-kindled rats have a significant reduction (*p* < 0.01) in seizure score after administration of Q808 (Figure 2A). The latency to seizure was significantly increased (*p* < 0.05) in the Q808-treated group compared with the PTZ model group (Figure 2B). All results indicated that Q808 showed an effective anti-convulsant activity against chronic PTZ-kindled seizure rats.

### DEGs in the Hippocampus and Hierarchical Clustering

**FIGURE 3:**
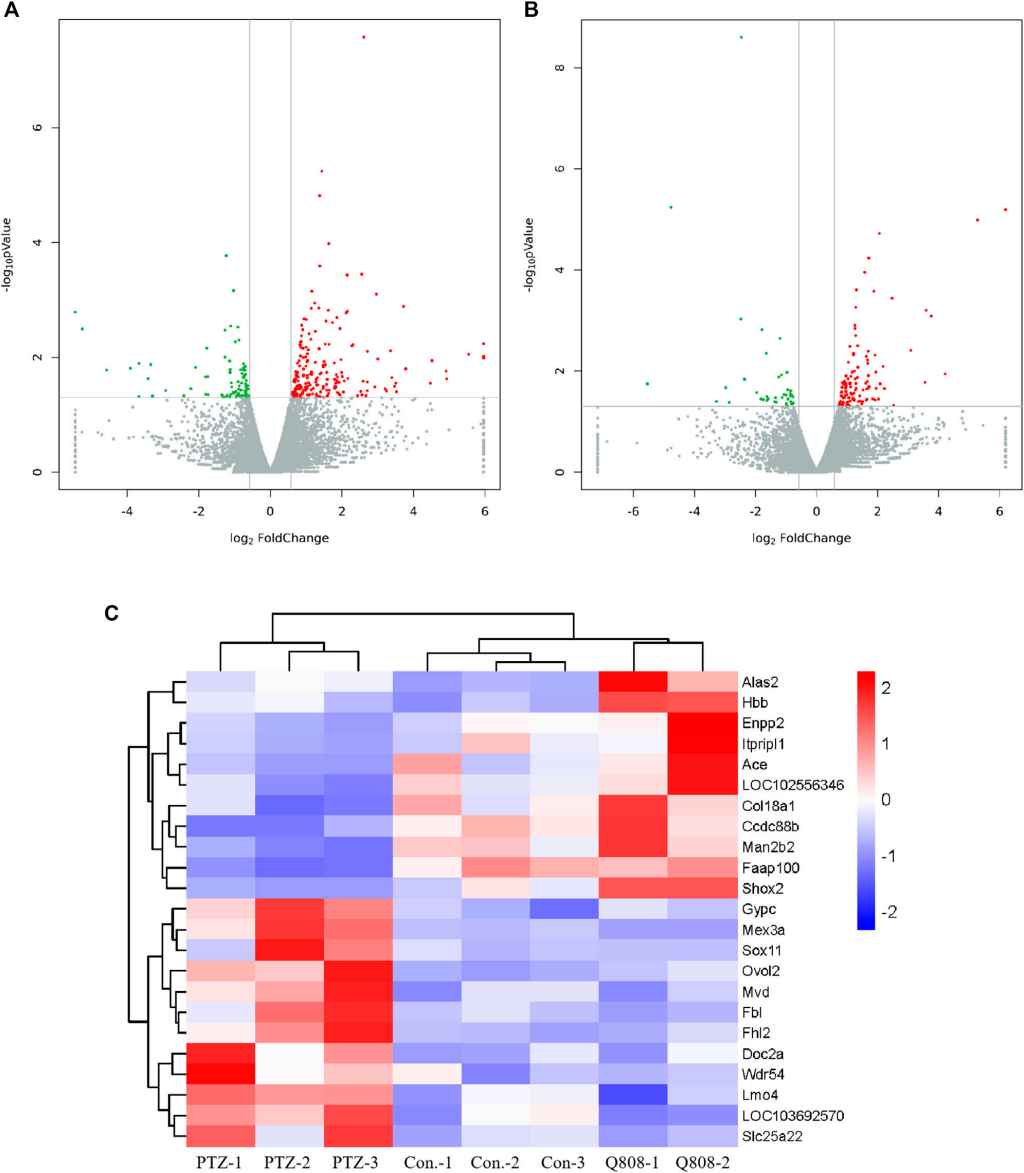
Volcano plot and heatmaps of hub genes in the hippocampus. **(A, B)** Volcano plot showing up-regulated (red dots) and down-regulated (green dots) DEGs and normal expressed genes (gray dots). **(C)** Heatmap showing the expression level of 23 hub genes across the three groups. Blue and red colors represent low and high levels of expression, respectively. Corresponding hierarchical clustering analysis of these hub genes is shown in black bars.

Total RNA was isolated from the hippocampus of rat and subjected to transcriptomic sequencing. The raw sequencing data can be found at GEO: GSE189785. The datasets of clean data are displayed in Supplementary Table S1 and showed that the medial clean data were 6.31 Gb per sample. In addition, the sequence alignment analysis indicated that the result of RNA sequencing was reliable because the ratio of clean reads was greater than 92.34% of Q30 and beyond 47.77% of the GC content. When compared to the Con. group, 289 genes were differentially expressed (82 down-regulated, 207 up-regulated) in the PTZ group (Figure 3A; Supplementary Table S2). Compared to the PTZ group, there were 152 genes differentially expressed in the Q808 group. Among them, 39 genes were down-regulated and 113 genes were up-regulated (Figure 3B; Supplementary Table S3). 23 out of 157 genes identified in the Con. and PTZ comparison were also identified in the Q808 and PTZ comparison and defined as hub genes (Table 2). The heatmap (Figure 3C) illustrates these alternate expression levels for all hub genes across the three groups. In the PTZ group vs. Con. group, the expression of 12 genes displayed an initial increase, but there was a decrease in the Q808 group vs. PTZ group; however, the expression of 11 genes exhibited a reduction in the PTZ group vs. Con. group, but there was a subsequent increase in the Q808 group vs. PTZ group. Then, the hierarchical clustering structure (Figure 3C) was determined based on the relative expression values of hub genes. The analysis showed that the Con. and PTZ groups had significantly different gene expression profiles, whereas the Q808 treatment group and Con. group had similar gene expression profiles. Therefore, after Q808 treatment, the gene expression levels of PTZ-kindled seizure rats could reverse to healthy controls.

**TABLE 2:** Information of 23 hub genes identified by RNA-seq analysis across the three groups.

Hub gene	PTZ vs. Con.		Q808 vs. PTZ	
	Log_2_FC	*p*-value	Log_2_FC	*p*-value
*Ace* (angiotensin I converting enzyme)	−1.136	0.049	1.918	0.005
*Alas2* (5′-aminolevulinate synthase 2)	1.110	0.030	1.123	0.027
*Ccdc88b* (coiled-coil domain containing 88B)	−0.868	0.031	1.119	0.021
*Col18a1* (collagen type XVIII alpha 1 chain)	−0.666	0.034	0.963	0.012
*Doc2a* (double C2 domain alpha)	1.006	0.002	−0.950	0.023
*Enpp2* (ectonucleotide pyrophosphatase/phosphodiesterase 2)	−0.703	0.014	1.639	0.023
*Faap100* (Fanconi anemia core complex-associated protein 100)	−0.840	0.016	0.839	0.045
*Fbl* (fibrillarin)	0.987	0.037	−1.326	0.041
*Fhl2* (four and a half LIM domains 2)	0.990	0.010	−0.969	0.049
*Gypc* (glycophorin C)	1.380	0.001	−1.003	0.030
*Hbb* (hemoglobin subunit beta)	0.776	0.026	1.303	0.001
*Itpripl1* (inositol 1,4,5-trisphosphate receptor interacting protein-like 1)	−1.107	0.044	2.032	0.036
*LOC102556346* (angiotensin-converting enzyme-like)	−0.978	0.005	1.696	0.004
*LOC103692570* (dihydropyrimidinase-related protein 5-like)	1.387	0.019	−4.768	0.001
*Lmo4* (LIM domain only 4)	0.709	0.018	−1.197	0.002
*Man2b2* (mannosidase, alpha, class 2B, and member 2)	−0.7607	0.017	1.040	0.007
*Mex3a* (mex-3 RNA binding family member A)	1.354	0.001	−1.790	0.002
*Mvd* (mevalonate diphosphate decarboxylase)	0.742	0.031	−0.938	0.036
*Ovol2* (ovo-like zinc finger 2)	2.552	0.001	−1.630	0.032
*Shox2* (short stature homeobox 2)	−3.674	0.012	5.274	0.001
*Slc25a22* (solute carrier family 25 member 22)	1.588	0.024	−2.363	0.015
*Sox11* (SRY box 11)	2.712	0.027	−3.280	0.040
*Wdr54* (WD repeat domain 54)	0.931	0.020	−1.067	0.029

### GO and KEGG Enrichment Analysis

**FIGURE 4:**
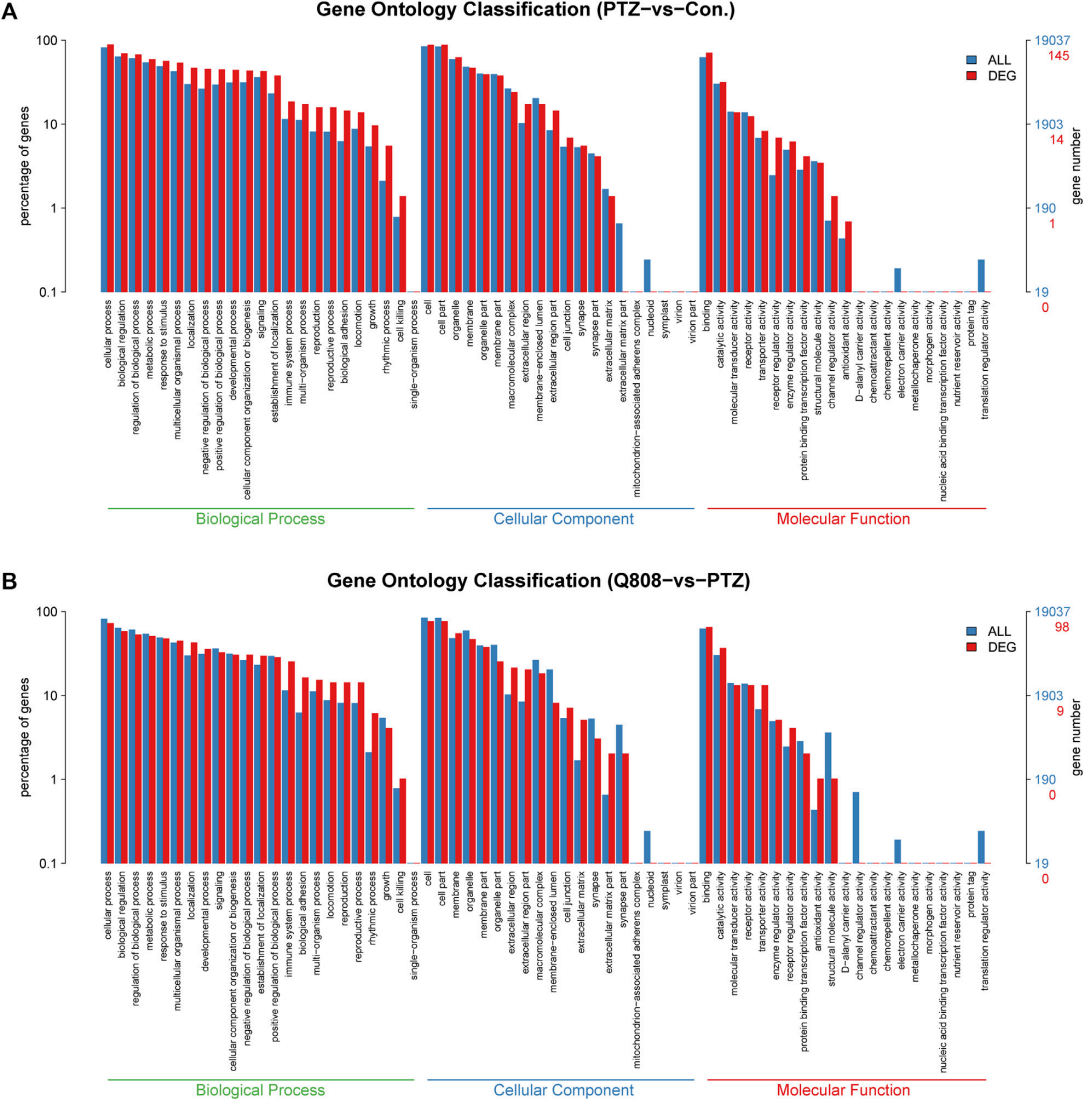
**(A)** GO classification of DEGs between PTZ and Con. groups. **(B)** GO classification of DEGs between Q808 and PTZ groups. The GO term of the most genes was cellular process, cell, and binding for the biological process, cellular component, and molecular function, respectively.

GO analysis and KEGG enrichment analysis were conducted to explore the biological functions of the DEGs. In the GO analysis, the number of DEGs annotated to GO functions was identified, and the enrichment of GO functional significance was covered. Between the Con. and PTZ groups, 2080 GO terms were significantly enriched (Supplementary Table S4). After treatment with Q808, 1070 GO terms were significantly enriched compared with the PTZ model group without Q808 treatment (Supplementary Table S5). For both groups, the GO term of the most genes was cellular process, cell, and binding for the biological process, cellular component, and molecular function, respectively (Figures 4A,B). The detailed information of the gene functions is shown in Supplementary Table S6, which suggests that most of the genes are correlated with neuro-apoptosis, neurogenesis, neuronal projections, and neurotransmitter regulation, such as neurotransmitter biosynthetic process, secretion, transporter activity, and receptor activity.

In KEGG pathway analysis between the PTZ and Con. groups, Q808 and PTZ groups revealed 35 and 33 pathways, respectively (Supplementary Table S7; Supplementary Figures S1, S2). After comprehensive analysis across the three groups, 21 significantly enriched signaling pathways were identified (Table 3), five of which, including glycine, serine, and threonine metabolism; ether lipid metabolism; renin–angiotensin system; renin secretion; and malaria pathways, were associated with hub genes and were regarded as hub pathways. The hub genes on these hub pathways were *Ace*, *Alas2*, *Enpp2*, *Gypc*, and *Hbb*.

**TABLE 3:** Twenty-one hub pathways across the three groups.

Term	ID	PTZ vs. Con.		Q808 vs. PTZ	
		*p*-value	Enrichment score	*p*-value	Enrichment score
VEGF signaling pathway	rno04370	0.002	5.985	0.032	3.581
Basal cell carcinoma	rno05217	0.002	5.593	0.036	3.346
Ether lipid metabolism	rno00565	0.008	4.944	0.021	4.437
Malaria	rno05144	0.012	4.135	0.000	14.844
Toxoplasmosis	rno05145	0.015	3.130	0.016	3.745
Graft-versus-host disease	rno05332	0.019	3.499	0.040	3.140
Amphetamine addiction	rno05031	0.020	3.446	0.041	3.092
Leishmaniasis	rno05140	0.021	3.394	0.042	3.046
Renin secretion	rno04924	0.022	3.345	0.044	3.002
Allograft rejection	rno05330	0.022	3.345	0.044	3.002
Asthma	rno05310	0.023	4.212	0.008	7.559
Bile secretion	rno04976	0.024	3.203	0.047	2.875
Focal adhesion	rno04510	0.029	2.309	0.003	4.144
Human papillomavirus infection	rno05165	0.030	2.001	0.006	2.993
Rheumatoid arthritis	rno05323	0.037	2.707	0.008	4.860
Fluid shear stress and atherosclerosis	rno05418	0.038	2.353	0.034	2.815
Renin–angiotensin system	rno04614	0.040	3.159	0.013	5.670
Viral myocarditis	rno05416	0.042	2.584	0.009	4.639
GnRH signaling pathway	rno04912	0.043	2.555	0.009	4.587
Glycine, serine, and threonine metabolism	rno00260	0.044	2.992	0.015	5.371
Bladder cancer	rno05219	0.048	2.843	0.016	5.103

### Validation of Candidate DEGs

**FIGURE 5:**
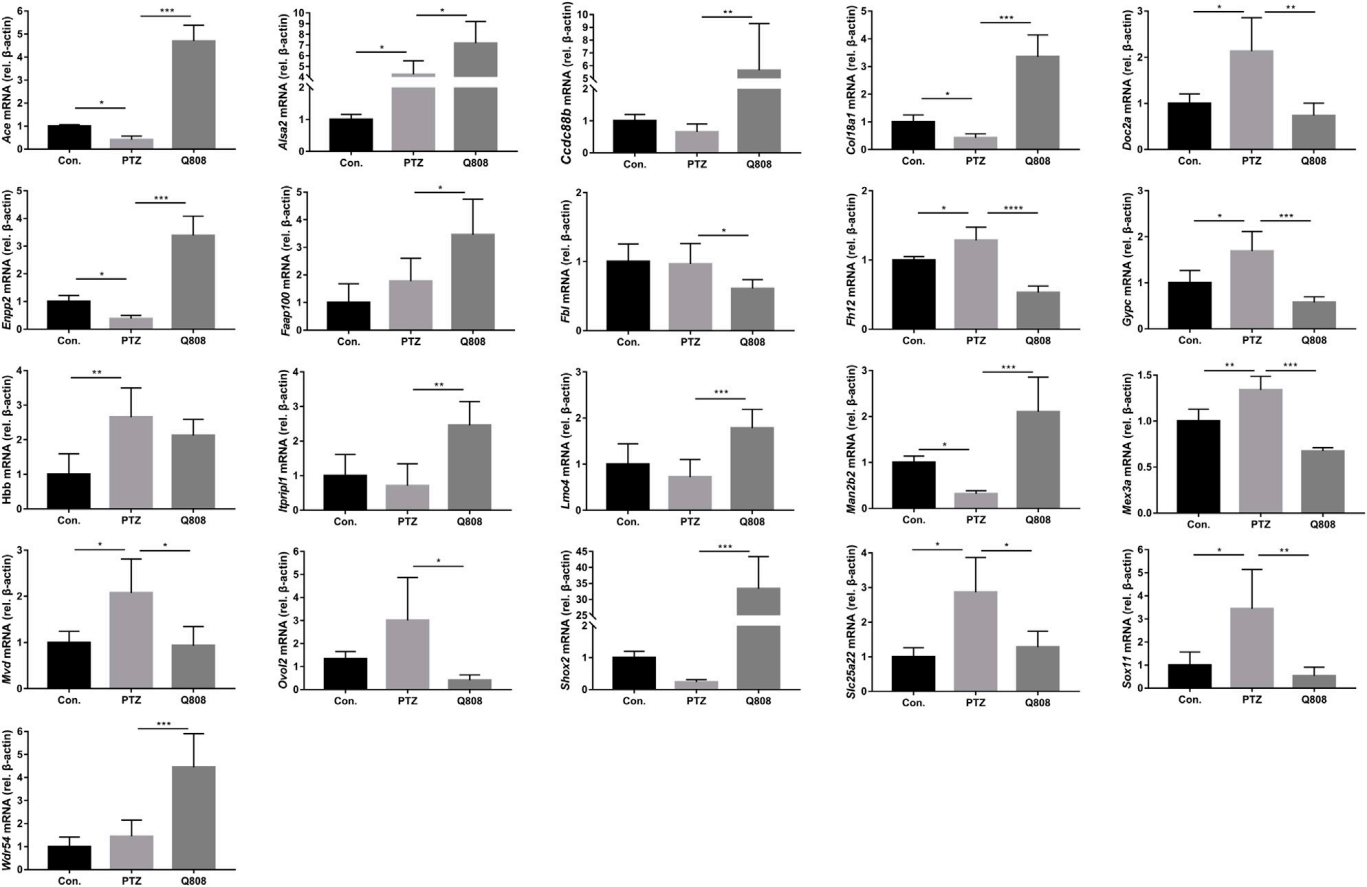
Altered gene expression in the hippocampus. Differentially expressed mRNAs which were identified in the RNA-seq data were further validated by qRT-PCR according to the fold-changes of qRT-PCR (2^−ΔΔCt^). *p < 0.05, **p < 0.01, and ***p < 0.001. Data are expressed as mean ± SD and analyzed using one-way ANOVA followed by Tukey’s test (*n* = 5).

qRT-PCR was conducted to verify the expression profiles obtained from RNA-seq and DEG analyses (Figure 5). We found that the expression levels of *Ace*, *Col18a1*, *Enpp2*, and *Man2b2* down-regulated significantly in the PTZ model compared with the Con. group. The mRNA expression levels of *Alsa2*, *Doc2a*, *Fh12*, *Gypc*, *Hbb*, *Mex3a*, *Mvd*, *Slc25a22*, and *Sox 11* were significantly up-regulated in the PTZ group compared with the Con. group. After Q808 treatment, the mRNA level of *Ace*, *Ccdc88b*, *Col18a1*, *Enpp2*, *Man2b2*, and *Shox2* increased by > 5-fold; the gene expression of *Alas2*, *Faap100*, *Itpripl1*, *Lmo4*, and *Wdr54* increased by one- to threefold; the expression of *Doc2a*, *Fbl*, *Fhl2*, *Gypc*, *Mex3a*, *Mvd*, *Ovol2*, *Slc25a22*, and *Sox11* remarkably decreased. Based on the RNA-seq results between the PTZ and Con. groups, the expression levels of *Lmo4* and *Ovol2* were significantly increased. However, when analyzing the qPCR results, no significant changes were observed. Furthermore, we found opposite changes in the qRT-PCR results compared with the RNA-seq results of the gene expression of *Faap100* between the PTZ and Con. groups and *Fbl*, *Lmo4*, and *Wdr54* between the PTZ and Q808 groups. The expression of *LOC102556346* and *LOC103692570* could not be determined because of the limited information of primers.

### Expression Level of GABA-Related Protein

**FIGURE 6:**
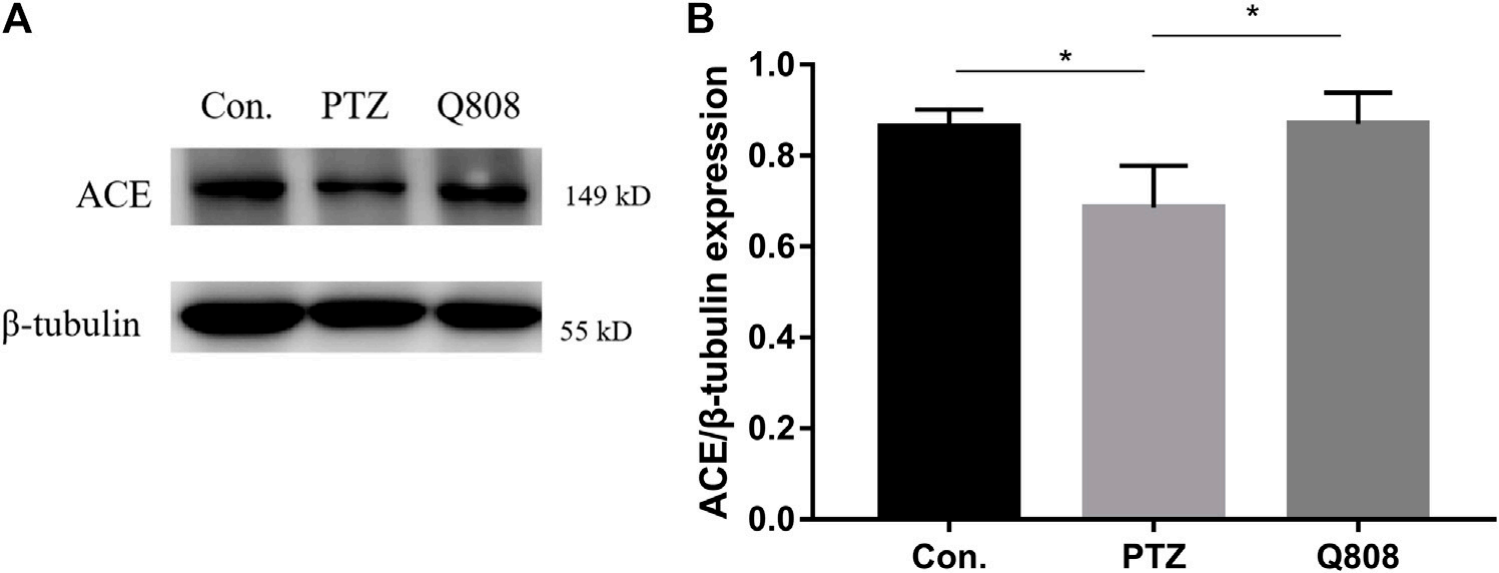
The protein levels of ACE in the hippocampus of rats were determined by Western blot analysis. **(A)** Representative Western blot bands of ACE. **(B)** Quantitative analysis of ACE. β-Tubulin was used as the internal control. *p < 0.05. Data are expressed as mean ± SD and analyzed using one-way ANOVA followed by Tukey’s test (*n* = 3).

According to the RNA-seq results and previous literature, one gene, namely, *Ace*, related to GABA release was identified, and the protein level was analyzed by Western blot (Figure 6). When compared with the Con. group, the expression levels of ACE in the PTZ group were significantly decreased (*p* < 0.05). Following Q808 treatment, the expression of ACE in the hippocampus significantly increased compared with that of the PTZ group (*p* < 0.05). The trend of ACE protein expression change was similar to the level of *Ace* mRNA expression.

## Discussion

This study showed the RNA-seq transcriptomic profile of the chronic PTZ-kindled seizure model in rats, which identified key transcriptomic signatures in PTZ-kindled seizure. When comparing Con. with the PTZ group, RNA-seq analysis of the hippocampal tissues identified 289 DEGs and revealed their functional enrichment. Experimental modeling and clinical studies have shown that seizures can cause neuronal death in the brain, and such brain injury may contribute to epileptogenesis (Henshall and Simon, 2005). After analysis of DEGs and their GO term, we identified some DEGs, such as *Bad*, *Ccl2*, *Kcnip3*, *Hmox1*, *Rras2*, *Grm8*, *Serpinf1*, and *Fbxw7*, which were associated with the cellular process such as regulation of neuronal apoptosis. Some of these genes have been reported, while some are not. Based on previous studies, *Bad* (*Bcl2*-associated agonist of cell death), an essential component of mitochondrial-dependent apoptosis, which increased sixfold in the PTZ group, has been reported in epilepsy by regulating cell death (Lu et al., 2021). The *Grm8* (glutamate metabotropic receptor 8) agonist has anti-convulsant activities, and it is recognized as a potential target for novel AEDs (Chapman et al., 1999). Considering that the mechanisms for neuro-apoptosis are not fully clarified and its important role in epileptogenesis, other genes identified in our study related to apoptosis may provide a new insight.

Apart from neuro-apoptosis, increased neurogenesis and neuronal projections were observed in seizures, and reducing epileptic neurogenesis and excessive projections could serve as a therapeutic approach to limit the occurrence of seizures and progression of epilepsy (Mo et al., 2019). Based on our RNA-seq results, some of DEGs with GO terms including neurogenesis and neuronal projections have been reported to be associated with epilepsy. For example, increased *Ace* (Almeida et al., 2012), *EphA10* (Xia et al., 2013), *Limk1* (Jiang et al., 2020), and *Sgk1* (Armas-Capote et al., 19912020) were associated with the seizure susceptibility. These genes were also significantly altered in our RNA-seq analysis, which could verify the accuracy of our sequencing results to a certain extent. We also detected a number of genes that were related to neurotransmitters between the PTZ and Con. groups. Imbalance of excitatory and inhibitory neurotransmitters may lead to epileptic seizures (Lascano et al., 2016). Expressional alterations of receptors and ion channels activated by neurotransmitters can lead to pathogenesis of epilepsy (Akyuz et al., 2021). Based on our results, the expression levels of neurotransmitter signaling *Slc17a8*, *Stx1a*, *Th*, *Shisa8*, *Htr1f*, and *Grm8* were significantly changed in the PTZ group. Examining the function of these genes in epilepsy could be a crucial goal to explain the disruption of the excitatory and inhibitory balance in the brain. In addition, some DEGs identified in our research have not been reported in epilepsy so far, and further research on these genes may improve the identification of novel therapeutic targets for treating PTZ-kindled seizure.

Our pathway analysis of genes between the PTZ and Con. groups showed that 59% of DEGs were linked to signal transduction pathways. During epilepsy, signal transduction mediated by several neurotransmitters, including serotonin, glutamate, and GABA, and inflammation of the central nervous system (CNS) could affect seizure severity and lead to some comorbidities such as mood disorders (Rocha et al., 2014). The comprehensive understanding of the underlying mechanisms is still in its infancy. The DEGs clustered in these pathways may provide comprehensive understanding of the underlying mechanisms.

Furthermore, we analyzed the transcriptomic profile across the three groups, namely, Con., PTZ, and Q808. Q808 is an innovative anti-convulsant chemical, which is currently under preclinical study. To date, no studies have characterized the gene expression response to Q808. After RNA-seq analysis across the three groups, 23 hub genes and 21 pathways were identified. Hierarchical clustering analysis of hub genes showed that changes in gene expression caused by PTZ can be reversed after Q808 treatment. qRT-PCR validation of hub genes generally supports the results obtained from the RNA-seq experiments. Through several literature searches, we found 10 hub genes were related to epilepsy (Table 4). Thus, it has a great possibility that Q808 exerts anti-convulsant activity by affecting these genes. Further analysis indicated that one gene, *Ace*, might be involved in modulating GABA levels in the hippocampus, and it was mediated by Q808. ACE converts angiotensin (Ang) I into Ang II and subsequently into Ang-(1–7) by ACE2 (Ramos, 19792021). Ang-(1–7)-induced GABA release in the striatum has been verified by Stragier’s team (Stragier et al., 2005). However, the effect of Ace on the GABA release in the hippocampus has not been reported. ACE is also one of the key components of the renin–angiotensin system (RAS) (Krasniqi and Daci, 2019), and the RAS is one of the hub pathways identified in this research. The RAS can modulate different neurotransmitter systems, thereby controlling the excitability of the brain (Albrecht et al., 1997). Considering that Q808 can increase the level of GABA (Li et al., 2017), ACE or RAS might be one of the targets of Q808. Our future research will focus on the effect of ACE or RAS on GABAergic interneurons, especially GABA release, in the epilepsy model, and examine its molecular mechanism. In addition, evaluating the long-time safety of AEDs is of great importance (Gaitatzis and Sander, 2013). Thus, a future research study to investigate the impact of long-term treatment with Q808 on the genes and pathways in healthy animals could be an attractive study and is indispensable for Q808 clinical use.

**TABLE 4:** Hub genes related to epilepsy.

Gene	Relationship with epilepsy	References
*Ace*	Carbamazepine inhibits *Ace* and influences the pathogenesis of temporal lobe epilepsy	Almeida et al. (2012)
*Doc2a*	A novel Ca^2+^ sensor for synaptic transmission; increased in human and rat temporal lobe epilepsy	Hu et al. (2019)
*Shox2*	A transcriptional regulator of ion channels; loss of *Shox2* could alter firing frequency and thereby influence the susceptibility to seizure generation	(Yu et al., 19912021)
*Slc25a22*	Contributes to the genesis and control of myoclonic seizures	Molinari et al. (2005)
*Hbb*	Increased in cortical tissue samples from patients with mesial temporal lobe epilepsy	Guelfi et al. (2019)
*Fh12*	Associated with the *KCNE1* (minK) potassium channel; decreased in human mesial temporal lobe epilepsies	Jamali et al. (2006)
*Col18a1*	Encodes the collagen XVIII protein; inactivation of collagen XVIII results in improper neuronal cell migration	Ackley et al. (2001)
*Ovol2*	A key regulator of neural development; required for efficient migration	Mackay et al. (2006)
*Sox11*	A transcription factor; plays an important role in proliferation, maturation, and differentiation of newborn neurons	Faigle and Song (2013)
*Lmo4*	Regulates calcium-induced calcium release in central neurons and modulates synaptic plasticity in the hippocampus	Qin et al. (2012)

In summary, our study is unique as it utilizes RNA‐seq to assess the gene expression in the hippocampus among normal control rats, chronic PTZ-kindled seizure rats, and PTZ model rats after treatment with Q808. The transcriptomic changes influenced by Q808 in the hippocampus are evident. 23 hub genes and 21 hub pathways across the three groups are identified, which confirmed prior studies and provided additional potential mechanisms of epileptogenesis and therapeutic targets for Q808.

## Data Availability

The datasets presented in this study can be found in online repositories. The names of the repository/repositories and accession number(s) can be found at: https://www.ncbi.nlm.nih.gov/geo/query/acc.cgi?acc=GSE189785.
