# Supplementary figures and images for: Hippocampus RNA Sequencing of Pentylenetetrazole-Kindled Rats and Upon Treatment of Novel Chemical Q808
Source: Front Pharmacol. 2022 Mar 8;13:820508. doi: 10.3389/fphar.2022.820508 (PMC8957222; doi:10.3389/fphar.2022.820508)

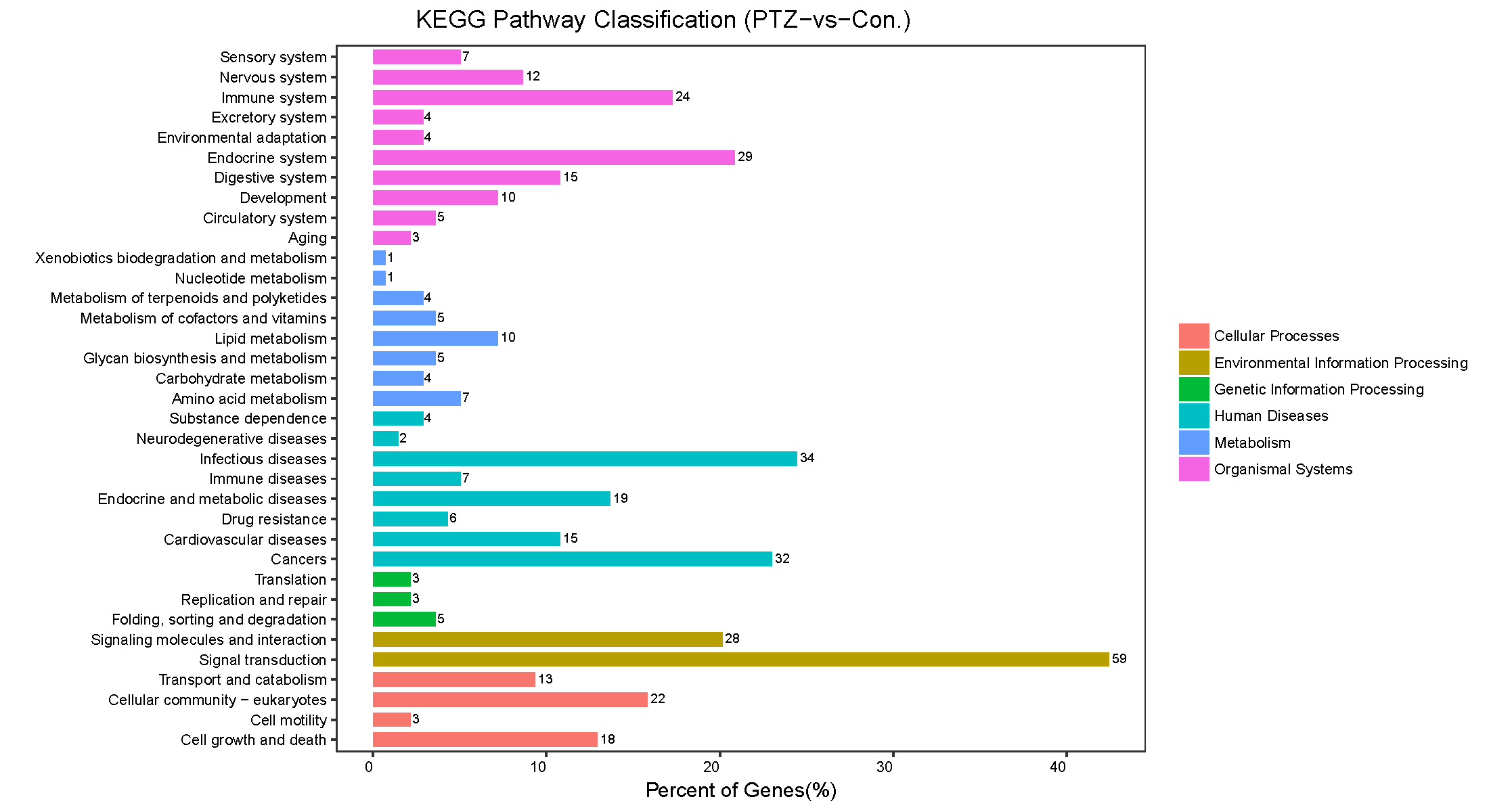

Supplement: Supplementary file 2 [file Image1.TIFF]

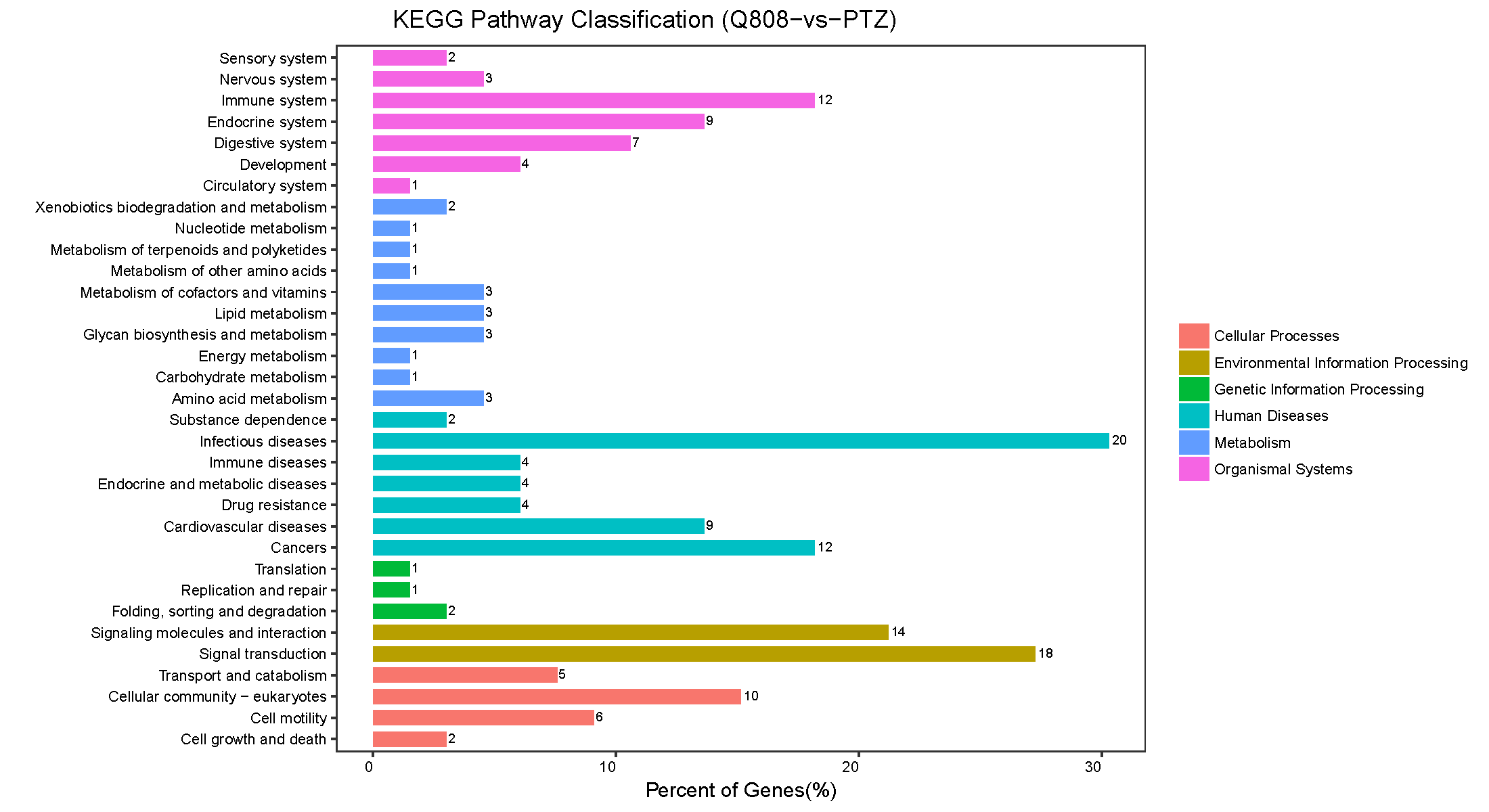

Supplement: Supplementary file 4 [file Image2.TIF]
